# Developing a new research tool for use in free-ranging cetaceans: recovering cortisol from harbour porpoise skin

**DOI:** 10.1093/conphys/cov016

**Published:** 2015-04-28

**Authors:** Thea Bechshoft, Andrew J Wright, Johan J Weisser, Jonas Teilmann, Rune Dietz, Martin Hansen, Erland Björklund, Bjarne Styrishave

**Affiliations:** af1 Department of Bioscience, Aarhus University, 4000 Roskilde, Denmark; af2 Faculty of Health and Medical Sciences, University of Copenhagen, 2100 Copenhagen Ø, Denmark

**Keywords:** Cetacean, cortisol, glucocorticoid, minimally invasive, skin, stress

## Abstract

We developed a chemical analytical procedure for sampling, extracting and determining epidermal skin cortisol concentrations (SCCs) in the harbour porpoise (*Phocoena phocoena*) using gas chromatography–tandem mass spectrometry. In brief, this involved a pressurized liquid extraction with a two-step solid-phase clean-up. A derivatization step was conducted prior to detection. To evaluate the new assay, cortisol was analysed in three different sample types obtained from four harbour porpoises: skin plates, dorsal fin skin plugs (with and without lidocaine) and epidermal scrapes. Skin cortisol concentrations could be measured using the new assay in the majority of the tested skin samples down to a minimal sample size of 49 mg dry weight (dw). Water content ranged from 10 to 46% in the plug samples, which had SCCs from 2.1 to 77.7 ng/g dw. Epidermal scrape samples had the highest water content (83–87%) and lower SCCs (0.6–15 ng/g dw), while the skin plates had intermediate water contents (60–66%) and SCCs of 2.6–13.0 ng/g dw. SCC was slightly higher in plugs with lidocaine than without (average values of 41 and 33 ng/g dw, respectively). Substantial within-individual variations in cortisol concentrations are also common in other matrices such as blood and hair. Some important factors behind this variation could be e.g. the animal's sex, age, body condition, reproductive stage, and the body region sampled, as well as season, moulting cycles and water temperature. Clearly, more research into SCCs is required. The findings described here represent the first critical steps towards using epidermal skin cell samples to assess chronic stress levels in cetaceans and the development of a widely applicable health-assessment tool in these species.

## Teaser

In this paper we present a new analytical method that allows the extraction of the stress hormone cortisol from naturally sloughed cetacean skin cells. Skin cortisol is unaffected by short-term cortisol fluctuations and could thus be used to assess chronic stress levels in cetaceans.

## Introduction

Marine mammals face numerous anthropogenic stressors, including chemical pollution, underwater noise and climate change, the combined consequences of which are often difficult to assess (Durner *et al.*, 2009; MMC, 2009; Wright, 2009; Sonne, 2010). Chronically stressful situations have been shown to affect the general state of marine mammal health in various ways; these include, but are not limited to, impaired immune function, increased mortality and reduced production of offspring (Fair and Becker, 2000; St Aubin and Dierauf, 2001; Sonne, 2010). In most mammals, cortisol is the dominant circulating glucocorticoid associated with a stress response and is typically measured in blood samples (St Aubin, 2001). However, baseline blood concentrations can be difficult to ascertain owing to short-term natural diurnal and seasonal fluctuations (Barrell and Montgomery, 1989; Suzuki *et al.*, 2003; Oki and Atkinson, 2004). Furthermore, the acute stress response associated with capture, handling and sampling, especially in wild animals, is itself reflected in the blood within minutes (Cook *et al.*, 2000; Eskesen *et al.*, 2009; Fair *et al*., 2014). The cortisol peak caused by such an acute stressor may potentially obscure baseline values or overshadow any effects on cortisol concentrations resulting from exposure to other (longer-term) stressors. These difficulties are enhanced in free-ranging marine mammals, where many species are simply too difficult or too large to capture or handle. Consequently, several forums have identified the pressing need for more suitable methods of assessing stress responses, and chronic stress in particular, in marine mammals (e.g. Wright *et al.*, 2007; ONR, 2009).

This has led to a growing interest in several promising new cortisol matrices in marine mammals, including vibrissae, hair, urine, saliva, baleen and faeces (Constable *et al.*, 2006; Pedernera-Romano *et al.*, 2006; Bechshøft *et al.*, 2012; Rolland *et al.*, 2012; Fair *et al.* 2014; Hunt *et al.* 2014). Whale blow may also be a potential candidate (Hogg *et al.*, 2009; Tizzi *et al.*, 2010; Dunstan *et al.*, 2012). Keratinous materials, such as vibrissae and hair, have been shown to be feasible indicators of chronic stress responses (Cook, 2012). Unfortunately, these two matrices are of little use when assessing chronic stress in cetaceans, because these species have little (if any) hair as adults. However, all other currently available minimally invasive matrices, such as faeces, may only be used to assess short-term cortisol fluctuations over extended time periods (Terio *et al*., 1999; Suzuki *et al*., 2003; Pedernera-Romano *et al*., 2006; Desportes *et al*., 2007; Sheriff *et al*., 2009, 2010; Schmitt *et al*., 2010; Funasaka *et al*., 2011; Cook, 2012). Unfortunately, the collection of such samples from animals in the wild is opportunistic at best (e.g. Rolland *et al.*, 2012). Thus, no reliable methods currently exist for assessing cortisol at baseline levels or over the longer term in free-ranging cetaceans. Such methods would preferably involve matrices that reflect chronic, not acute, cortisol concentrations and, ideally, also be minimally invasive to avoid handling/sampling artifacts.

Considering this, expert discussions have highlighted skin (epidermis) as a potential alternative hormone matrix (e.g. Wright *et al.*, 2007; ONR, 2009). Cetacean epidermal cells have previously been used for genetic, diet and contaminant studies (e.g. heavy metals and trace elements; Hunt *et al.*, 2013; Savery *et al.*, 2013; Browning *et al.*, 2014), but never for assessment of hormones. Hormones have, however, been found in cetacean blubber (Trego *et al.*, 2013; Trana *et al.*, 2015), a matrix closely associated with skin.

Cortisol released into the bloodstream following an acute stressor (e.g. capture) would not be anticipated to enter the skin matrix for days or even weeks (Hicks *et al.*, 1985; Browning *et al.*, 2014). Thus, cortisol in cetacean skin is expected to reflect levels of chronic stress. Validation of this statement is still required, but if found to be true, skin samples could provide information on long-term physiological status in cetaceans in terms of chronic stress. Furthermore, cetacean skin samples can be obtained in non- or at least minimally invasive ways (Noren and Mocklin, 2012). In fact, cetacean skin samples for DNA analyses have already been collected non-invasively and without direct handling of the animal using green kitchen scourers, although with mixed success (Harlin *et al.*, 1999; but see also Farro *et al*., 2008).

Accordingly, the main aim of this study was to develop a chemical analytical procedure for sampling, extracting and determining epidermal skin cortisol concentrations (SCCs) in the harbour porpoise (*Phocoena phocoena*) by use of gas chromatography–tandem mass spectrometry (GC-MS/MS).

## Materials and methods

### Samples

#### Bycaught, dead animals

**Figure 1:**
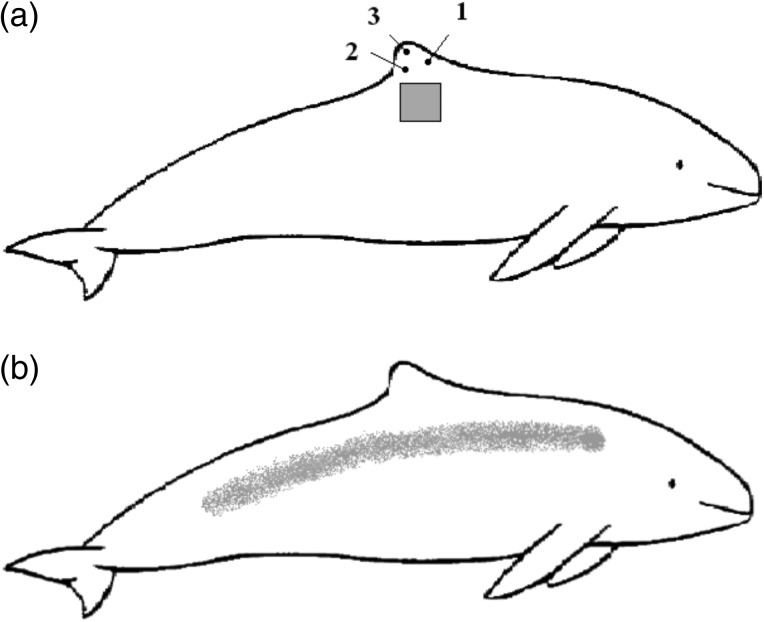
(**a**) Outline of a harbour porpoise (*Phocoena phocoena*) with indications of sampling locations of skin plates and biopsy plugs taken from dead bycaught animals in Danish waters and used in the development of the skin cortisol analysis method presented in this study. The shaded square area indicates the location of the skin plate, 10 cm × 10 cm in size. Points 1–3 indicate the approximate locations of the 6 mm biopsies. (**b**) Outline of a harbour porpoise (*P. phocoena*) indicating where alongside the animal's left and right side non-invasive skin sampling devices were used to collect samples of sloughed skin cells. (Porpoise outline adapted from the Wikimedia Commons file ‘Harbor_porpoise_size.svg’.)

Skin samples (epidermis) were collected from two dead harbour porpoises (ID 43720, a subadult female, and ID 43721, an adult female; Table 1) that were accidentally bycaught in gill nets in Danish waters, frozen within a day of death and kept frozen at −20°C for 12 or 18 months (for IDs 43720 and 43721, respectively) until 24 h before sampling. After collection, the skin samples were returned to −20°C for 13 months until analysis. The samples collected were a 10 cm × 10 cm skin plate, taken from the right side of each animal just below the dorsal fin, and three 6 mm dorsal fin biopsies (Fig. 1a), taken as if the animal was to be pinned for tagging in the wild (Teilmann *et al.*, 2007; Sonne *et al.*, 2012). Published methods were followed for the pin biopsies (Teilmann *et al.*, 2007; Sonne *et al.*, 2012), except that the analgesic lidocaine (5%) was applied only to the left side of the dorsal fin before cork boring to allow for comparison with wild tagged animals as well as a preliminary assessment of any possible effects of the application. The skin plate was primarily used for assay development, while the plugs were used to assess the potential value of such biopsies to future SCC studies.

**Table 1:** Skin cortisol concentration (in nanograms per gram dry weight) as measured in skin samples from harbour porpoises (*Phocoena phocoena*)

ID no.	Sex	Standard length (cm)	Date (circumstance)	Sample type	Sample location	Sample wet weight (mg)	Sample dry weight (mg)	Water content (%)	Cortisol concentration (ng/g dw)
43720	Female	117	February 2011 (bycaught in gill net, dead)	Skin plate	Right	900	306	66	13.0 ± 1.5^a^
				Plugs (clean)	Right 1	91	49	46	2.1
					Right 2 + 3	164	111	32	2.2
				Plugs (with lidocaine)	Left 1	87	66	24	LOD^b^
					Left 2 + 3	174	97	44	4.7
43721	Female	154	August 2010 (bycaught in gill net, dead)	Skin plate	Right	670	268	60	2.6
				Plugs (clean)	Right 1	71	64	10	70.1
					Right 2 + 3	162	106	35	57.7
				Plugs (with lidocaine)	Left 1	50	34	32	LOD^b^
					Left 2 + 3	132	99	25	77.7
47501	Male	151	April 2012 (bycaught in pound net, alive)	Scraper	Right	701	89	87	0.6
					Left	869	112	87	NA^c^
2012/60022	Male	144	August 2012 (bycaught in pound net, alive)	Scraper	Right	414	72	83	15
					Left	629	96	85	2

Abbreviations: dw, dry weight; LOD, limit of detection; NA, not available. ^a^±1.5 corresponds to 11.6% (relative standard deviation). ^b^Signal-to-noise ratio <3. ^c^Missing internal standard.

#### Bycaught, live animals

Prior to commencing this project, several non-invasive skin sampling techniques had been tested on nine free-ranging harbour porpoises caught in pound nets in Danish waters and handled in the ongoing tagging project there (Teilmann *et al.*, 2007). Sampling involved simply running one of the various sterilized collection devices along each side of each animal once using single, not overly forceful strokes that were easy to replicate (Fig. 1b). The devices tested were as follows: (i) a green kitchen sccourer; (ii) a plastic laboratory tube; (iii) P40 and P80 grit emery cloth; and (iv) a rubberized ice scraper (Supplementary material Fig. 1). For reasons discussed in detail in the results section of the paper, the ice scraper was the only one of these tested devices to yield sufficient epidermal skin samples. The collected samples ultimately used here were transported on dry ice (−70°C) and stored in a −20°C freezer for 8 or 11 months (for IDs 2012/60022 and 47501, respectively) before analysis. No plugs taken for the purpose of tagging were used in this study.

#### Application

To evaluate the new assay, cortisol was analysed in three different skin samples: those obtained by the minimally invasive use of a rubberized ice scraper; the dorsal fin plugs taken during simulated tagging procedures on lethally bycaught animals (with or without prior application of lidocaine); and the skin plates, also from the lethally bycaught animals. Sample size [dry weight (dw)] and water content of these samples are shown in Table 1. Plug 1 was analysed on its own, while plugs 2 and 3 were pooled to test for potential effects of an increased sample size (lidocaine-covered samples and clean samples were analysed separately).

### Cortisol analysis

The method developed was initially based on the work of Hansen *et al*. (2011a), in which three steroid hormone classes were extracted and analysed from environmental matrices (i.e. soil and animal manure). We believed this method to be adaptable because we expected that cortisol would have similar physicochemical properties to those steroid hormone classes and that cetacean skin would have a high lipid content, much like the environmental matrices analysed.

#### Chemicals

Cortisol with an analytical purity >96% was used in calibration, optimization and method development studies. As a part of our quality assurance and quality control scheme, a derivatization quality control standard (DCS), 17β-estradiol-17-acetate (purity >99%), and an instrument control standard (ICS), estrone-3-methyl ether (purity >99%), were added to each sample. All three compounds were obtained from Sigma-Aldrich (Glostrup, Denmark). The deuterated analogue of cortisol, d_4_-cortisol (purity >98%; Toronto Research Chemicals, Ontario, Canada), was applied to all samples as an internal standard (IS). All organic solvents were of high-performance liquid chromatography grade, with a purity >99.5%. The derivatization reagents *N*-methyl-*N*-trimethylsilyl-trifluoroacetamide (MSTFA), *N*-trimethylsilylimidazole (TMSI) and 1,4-dithioerythritol (DTE) were obtained from Sigma-Aldrich (Glostrup, Denmark). The derivatization mixture consisted of 1000 μl MSTFA, 50 μl of a 40 µg µl^−1^ DTE pyridine dilution and 2 μl TMSI. Cortisol, IS, DCS and ICS were all dissolved in methanol in separate volumetric glass flasks to produce stock solutions of ∼100 ng µl^−1^. Moreover, working dilutions in methanol were prepared from these stock solutions. Helium was used as the carrier gas and argon as the collision gas in the GC-MS/MS process.

#### Sample preparation

Method development was performed using 10 cm × 10 cm skin plates, a size chosen to ensure sufficient material for the exploratory use. In skin plate and plug samples, only the 2 mm top layer of epidermal cells was used to ensure that no blubber was included in the analysis. For the scrape samples, the epidermal cells were used as they were collected. Water content and dry weight were then determined by weighing samples before and after 24 h of lyophilization using a freeze dryer (FD3; Heto Lab equipment, Allerød, Denmark) operating at −48°C and 1 Pa (10^−5^ bar).

#### Pressurized liquid extraction procedure

As mentioned above, the pressurized liquid extraction (PLE) extraction procedure used was adapted from a method for measuring cortisol in animal manure and agricultural soil (Hansen *et al*., 2011a), using the ASE 200 PLE system (Dionex, Sunnyvale, CA, USA). Prior to extraction, 22 ml PLE stainless-steel cells were packed with 1 g activated silica gel (particle size 0.063–0.100 mm mesh; Merck, Darmstadt, Germany) and 1 g diatomaceous earth (DE; Dionex). Furthermore, sample tissue was mixed with 1 g activated DE and ground in a mortar before being applied to the cell. Silica gel and DE were activated for 24 h at 105°C before use. A single cellulose filter (size 19.8 mm; Dionex) was applied at the bottom and at the top of the cell. Cortisol was extracted using a methanol and acetone mixture (1:1, v/v) at 1500 psi and 80°C with a 5 min preheat followed by two 5 min static extraction cycles with 60% flush volume and 120 s purge period.

#### Cleaning up of extracts

The crude PLE extracts were subjected to a two-step clean-up process to reduce matrix components that may interfere with the sensitive GC-MS/MS system (Hansen *et al*., 2011b).The first step used an amino-propyl column (NH_2_, 500 mg amino-propyl SPE cartridge; Waters Sep-pak, Dublin, Ireland), which had been preconditioned twice, each time using 2 ml *n*-heptane, to remove fatty acids and phospholipids (Kaluzny *et al.*, 1985). The PLE extracts were evaporated close to dryness (∼1–2 ml left in the vials) at 60°C under a gentle stream of N_2_ with circular motions. Two millilitres of isopropanol was added to the vial and evaporated close to dryness a second time. After this, a second aliquot of 2 ml of isopropanol was added, and the mix once again evaporated close to dryness, before 100 μl CHCl_3_ was added to the vials and the extracts transferred to the preconditioned NH_2_ column. The vials were then washed with 200 μl CHCl_3_:isopropanol (2:1,v/v), to ensure a quantitative transfer of the analyte from the vials to the column. Finally, the steroid hormones were re-extracted from the column using 5 ml CHCl_3_: isopropanol (2:1, v/v).

The second clean-up step for separating the steroid hormones from lipids (such as mono-, di- and triglycerides, sterols, stanols and cholesterols) required a silica gel SPE column consisting of a 3 ml glass column (LiChrolut; Merck, Darmstadt, Germany), a cellulose filter and 1 g activated silica gel soaked in *n*-heptane (Hansen *et al.*, 2011a, b). The extracts from the first step were evaporated to dryness and reconstituted in 50 μl CHCl_3_ and 450 μl *n*-heptane before being loaded into the silica gel column. The vials were washed with 500 μl *n*-heptane to ensure quantitative transfer. The gel columns were then flushed with 5 ml *n*-heptane followed by 10 ml *n*-heptane:acetone (90:10, v/v). Hereafter, androgens, estrogens and progestagens were eluted with 5 ml *n*-heptane:acetone (65:35, v/v; Hansen *et al.*, 2011a). One final flush in 5 ml methanol was used to extract the more polar hormones, such as the corticosteroids, including cortisol.

#### Derivatization

The purified extract was evaporated to dryness and transferred to a GC-vial with a 300 μl insert using two times 100 μl methanol. Next, 100 μl 0.2 ppm DCS solution was added, and the purified extract was evaporated to dryness using N_2_ as described in the previous subsection. The steroid hormones were derivatized using 50 μl derivatization reagent mixture (1000 μl MSTFA, 50 μl DTE dilution and 2 μl TMSI) at 60°C for 1 h. The extract was then reconstituted in 200 μl 0.1 ppm ICS *n*-heptane solution (Hansen *et al.*, 2011a).

#### Gas chromatography–tandem mass spectrometry

A Bruker Scion™ TQ hyphenated system (EI interface; Bruker Daltonik, Bremen, Germany) with a GC PTV-injector and a Zebron-5HT Inferno column (30 m × 0.25 mm, 0.25 μm; Phenomenex Inc., Torrance, CA, USA) was used to determine SCC. A sample size of 10 μl was injected with a solvent-split/splitless PTV-program starting at 120°C and kept in the injector for 0.30 min (glass split liner with a glass frit; Varian Inc., Palo Alto, CA, USA) before it was subjected to a temperature ramp of 200°C/min to 325°C, where the temperature was held for 11.50 min. The column oven temperature programme was set at 150°C for the first 2 min, before increasing the temperature to 230°C (25°C/min); this temperature was maintained for 3.7 min. The total run time was 30 min, during which a constant carrier gas flow of 1.0 ml/min helium was applied. The triple-quadrupole mass spectrometer was operated in positive electron ionization mode using selected reaction monitoring mode with argon as the collision gas. The optimized ion transitions and collision energies were 578.4 > 331.1 (collision energy 10 V), 578.4 > 374.2 (collision energy 10 V) and 578.4 > 432.2 (collision energy 30 V) for cortisol. The IS, d_4_-cortisol, yielded two transitions: 582.4 > 311.1 (collision energy 18 V) and 582.4 > 378.4 (collision energy 5 V). A reference chromatogram is displayed in Supplementary material Fig. 3.

The assay described above was then subjected to a PLE optimization and recovery experiment, to determine absolute recovery and relative recovery rates, as well as a standard addition experiment (see Supplementary material). The primary reason for using GC-MS/MS instead of a radioimmunoassay when measuring SCCs was the hope that the newly developed method can later be expanded to cover more glucocorticoid hormones (which would not all be measurable by using radioimmunoassay). In addition, this method should avoid the cross-reactivity that is frequently observed when using radioimmunoassays.

### Method validation

Initially, the absolute recovery of each step in the method was found, followed by a determination of full assay absolute and relative recovery using a pre- and postspike approach. Full assay and instrument variation were based on the d_4_-cortisol peak area. Experimental details of this as well as of the GC-MS and derivatization procedure, the PLE-optimization and recovery experiment and the standard addition tests can be found in the Supplementary material.

## Results

### Method validation

We aimed to obtain an optimal 100% absolute recovery when analysing skin samples, but were able to achieve only 20.6% for cortisol and 20.1% for the internal standard (d_4_-cortisol) when spiked at 100 ng per sample. While not ideal, this is a sufficient recovery rate for use in a new analysis method with no other contemporaries. In any case, and more importantly, we found an impressive relative recovery rate of 102.4 ± 10.5% when using d_4_-cortisol as an internal correction standard for cortisol (Supplementary material).

In the standard addition experiment, the native cortisol concentration was determined in triplicate from a homogenized sample, subdivided into three aliquots (sample sizes of 306 ± 3 mg dw). The endogenous concentration of cortisol was 13.0 ng/g, with an assay repeatability of 11.6% (relative standard deviation, *n* = 3). Furthermore, one sample was injected three times to determine instrument repeatability for cortisol and d_4_-cortisol. The peak area variations (relative standard deviation) were 2.7 and 1.7% for cortisol and d_4_-cortisol, respectively, in the skin samples containing 306 ± 3 mg dw. Given that no skin reference material was available, the limits of detection (LODs) were evaluated visually based on signal-to-noise ratios. The instrument LOD and limit of quantification (LOQ) in neat standards were calculated using a cortisol calibration curve with a linier dynamic range of a cortisol standard curve from 2.5 ppb to 2.5 ppm and a *r*^2^ of 0.9978. The calculated instrument LOD and LOQ were 37 and 113 ppb, respectively (Araujo, 2009).

Of the devices tested, only the ice scraper consistently collected enough material (sample size of ≥49 mg dw, *n* = 4, two each from two tagged male porpoises, ID 47501 and ID 2012/60022; Table 1) that could be easily extracted for the ensuing analysis. The samples collected via the other techniques were more variable in size (and would thus require multiple strokes to be assured of reaching the critical sample size of ≥49 mg dw) and, with the exception of the plastic tube, harder to extract from the collection devices.

### Analysis results

Cortisol could be measured using the new assay in 79% of the tested skin samples, reliably down to the limit seen in this study of 49 mg dw in one clean plug sample (14 samples in total, out of which two were below the LOD and one was not available owing to missing internal standard; see Table 1). The instrument control standard deviated <10% within batch, and the derivatization reaction was to completion (99% or higher) in all skin samples. The signal-to-noise ratio of d_4_-cortisol was >30 in every sample except for one (47501 left), where d_4_-cortisol could not be detected. Water content in the plug samples ranged from 10 to 46% and the SCCs from 2.1 and 77.7 ng/g dw. The skin scrape samples had the highest water content (83–87%) and lower concentrations of cortisol (0.6–15 ng/g dw), while the two skin plates had water contents and SCCs of 60 and 66% and 2.6 and 13.0 ng/g dw, respectively. Measured SCCs were slightly higher in plugs with lidocaine than without, with average values of 41 and 33 ng/g dw, respectively.

## Discussion

Cortisol concentration could be determined in all plug samples of sufficient size (≥49 mg dw), including those treated with lidocaine. Use of the rubberized ice scraper provided sufficient sample material for the assay as described here. Accordingly, it is clearly possible to obtain samples from any smaller cetacean already in hand, for example in connection with tagging, disentanglement efforts or health assessments (although it should be noted that collection of skin prior to direct handling at the sample site is advised because direct handling may result in loss of sloughed skin cells and thus a considerably smaller scraper sample). Although remote skin collection was not attempted here, it may also be possible to collect sufficient samples of skin naturally sloughed by larger cetaceans at sea (Harlin *et al.*, 1999; Engelhaupt, 2004). Skin biopsy punches from tissue banks could also be used as source material, which would enable retrospective temporal studies. Cortisol has proved highly stable in other keratinous matrices (Bortolotti *et al.*, 2009; Warnock *et al.*, 2010; Webb *et al.*, 2010), so it is entirely possible that even relatively old frozen skin samples (even if lipokeratinocytic in nature; Menon *et al.*, 1986; Pfeiffer and Rowntree, 1996; Reeb *et al.*, 2007) could provide useful analysis material.

Considerable differences in measured SCCs were observed both between and within sample types (Table 1). However, substantial differences in inter-individual cortisol concentrations are commonly observed in other matrices, such as blood and hair (Tryland *et al.*, 2002; Haave *et al.*, 2003; Davenport *et al.*, 2006; Suavé *et al.*, 2007; Eskesen *et al.*, 2009). Although we do not know whether this is the case in the present study, such differences can be dependent on factors such as the animal's sex, age, body condition, reproductive stage and which body region is sampled, as well as season, moulting cycles and water temperature (St Aubin *et al.*, 1990; Suzuki *et al.*, 2003; Macbeth *et al.*, 2010; Funasaka *et al.*, 2011).

Water content in the samples may also play a role. Differences between the scrape samples were already visible to the naked eye during the clean-up process of the analysis (Supplementary materialFig. 2), with the colour of these samples ranging from clear to yellow and green. This difference in colour was not observed between the plug samples. It is also possible that the presence of algae (or other interfering compounds) may potentially be increasing the weight of the sample and thus influencing the concentration calculation. Although the low sample size should again be kept in mind here, this could be another potential factor in the observed variation in scrape SCCs.

As evident in Table 1, a single clean plug from a sampled animal yielded enough material to provide a cortisol reading. However, this was not the case for the single lidocaine-treated plugs. Whether this was due to the lidocaine or to the lower sample weight of the lidocaine-treated plugs remains to be resolved. It was not possible to fully determine whether lidocaine application compromised the assay. However, the finding that the SCC values obtained from the various plug samples for any given animal were much closer to each other than to the SCC value of that animal's skin plate suggests that the analgesic had little, if any, effect.

Another possible cause of some of the observed differences is that the plugs were from dead animals, while the scrapes were from live animals. However, given that the concentrations in the skin plate were closer to the flank scrapes (Table 1), we believe that it is more likely that the differences observed are due to natural variation between body regions (keeping in mind that there could also potentially be variations in cortisol over the length of the flank scrape itself). Such intra-individual differences between body regions have also been reported elsewhere and are likely to relate to diversity in physiological parameters, such as epidermal cell growth rates, degree of vascularization and variations in epidermal thickness (Colbert *et al.*, 1998; Bryan *et al.*, 2010; Miller *et al.*, 2011). Choice of cetacean body region(s) to be sampled in further SCC studies should thus be considered carefully.

The present study describes an analytical method for quantifying cortisol in harbour porpoise epidermal skin cells. In addition, it documents that cortisol concentrations can be assessed in a range of different harbour porpoise skin samples. Based on the methodology described here, ensuing studies should investigate questions such as whether other hormones can be measured in cetacean skin and whether SCC is related to other hormones, such as aldosterone, which also appears to be of importance in the cetacean stress response (Thomson and Geraci, 1986; St Aubin and Geraci, 1990; Fair *et al.*, 2014). Another question is whether SCC reflects only adrenal activity or whether some of the cortisol is locally derived (as has been shown in other mammals; Slominski *et al.*, 2013). This will require, at least in part, an exploration of the relationship between blood cortisol concentration and SCC. A study by Trego *et al.* (2013) lends credibility to the existence of biologically relevant concentrations of hormones in skin as their study linked progesterone in blubber, a matrix very closely associated with skin, with pregnancy in four different species of cetaceans.

Besides these questions, the essential next step in the use of SCCs is a physiological validation to determine the biological relevance of the SCC values obtained and how long the hormonal response to an acute stressor takes to get into, and persist within, the skin matrix. Importantly, there is also a need to understand how hormones carried in the bloodstream are incorporated into the skin matrix; by diffusion or by simple cell renewal. Browning *et al.* (2014) found that isotope signatures took 14–23 days before their signal was detectable in the epidermis of bottlenose dolphins, while Hicks *et al.* (1985) found the skin cell renewal rate in bottlenose dolphins to be somewhat slower than that, with 99% of the [6-^3^H]-thymidine-labelled skin cells reaching the epidermis by day 22 after injection.

In addition, the relationship between blood cortisol concentrations and cortisol concentrations measured in the skin samples must be assessed, and basal cortisol concentrations and any inter- or intra-individual fluctuations must be evaluated. Finally, as discussed above, studies of the potential influence on SCC of basic variables, such as body region, sex, age, reproductive status and season, are also required in order to assess the further uses of cetacean skin in stress-related research. The present study provides a basis for these further investigations. Despite the outstanding questions, the findings described here represent the first critical steps towards the use of epidermal skin to assess chronic stress levels in cetaceans. The potential use of cortisol obtained from cetacean skin samples as a measure for chronic stress in these species has widespread management implications. As managers gain greater appreciation of the role of stress responses in mediating cumulative impacts, the potential exists for SCC to become a powerful tool for monitoring the general health of various cetacean populations.

## Supplementary material

Supplementary material is available at *Conservation Physiology* online.

## Funding

The analyses presented here were funded in part by the Office of Naval Research [grant no. N000141210896], the Aage V. Jensen Foundation, Humane Society International and OceanCare. The authors thank each organization for their interest and support.

## Supplementary Material

Supplementary Data
