# Supplementary material for: Developing a new research tool for use in free-ranging cetaceans: recovering cortisol from harbour porpoise skin
Source: Conserv Physiol. 2015 Apr 28;3(1):cov016. doi: 10.1093/conphys/cov016 (PMC4778458; doi:10.1093/conphys/cov016)
Supplement: Supplementary Data [file cov016supp.zip › cov016supp.docx]

**Supplementary material**

*Gas chromatography-tandem mass spectrometry and derivatization*

Cortisol with three trimethylsilyl groups was chosen as precursor ion that has a *m/s* of 578.4 amu. The product ions were 331.1 (quantifier ion), 374.2 (qualifier ion one), and 432.2 (second qualifier ion) with a retention time (RT) of 28.52 minutes. D_4_-cortisol had a mass of 582.4 amu, the product ions were 311.1 (quantifier ion) and 378.4 (qualifier ion), and RT was 28.51 minutes. Chromatograms of cortisol and d_4_-cortisol obtained from a plug sample (ID: 43721, plug 2 & 3) can be seen in Supplementary material Fig. 3.

*PLE-optimization and recovery experiment*

The absolute (AR) and relative (RR) recovery rates of the method were determined without skin matrix in the extraction cell. The experimental approach was a pre- and post-spike setup with a spiking level of 1,000 μL 0.1 ppm d_4_-cortisol solution in methanol. The analytical procedure was done as described in the main paper (under Matrials and methods). AR of cortisol and d_4_-cortisol were determined and the RR for cortisol to IS derived.

Initially, each step in the methodology was tested separately; the packed PLE cell, the amino-propyl column, and the silica gel column. The PLE cell contained 2 g DE, 1 g silica gel and 0.25 g graphite. The cortisol ARs were 85±4.5%, 86.8±10% and 55±8.8% (n=3) for the three steps, respectively. In the cortisol assay, in this present work, graphite was not included in the cell packing. The AR of the entire assay was 20.6±9.9% for cortisol (n=3) and 20.1±3.4% for d_4_-cortisol (n=3), with the RR between cortisol and d_4_-cortisol found to be 102.4±10.5%.

Consequently, it is possible to apply d_4_-cortisol to the sample prior to extraction as an internal standard to quantify for cortisol loss during the clean-up and for method variation. Furthermore, the method variation for d_4_-cortisol was 17% (SD%, 3 replicates) in samples without skin tissue and 15% (SD%, 9 replicates) in samples with skin plate tissue, indicating that the presence of co-eluting compounds from the skin tissue do not cause chromatographic problems throughout the method.

*Standard addition*

The AR and RR experiment was followed up by a standard addition experiment using skin samples of 300 mg dw. Only a single skin plate from one of the lethally bycaught animals was used for this experiment. Nine replicate samples of 306 ± 3 mg (dw) were packed in nine different PLE cells. Three cells were spiked with IS only to obtain native content of cortisol. Two cells were fortified with 5 ng (50 μL 0.1 ppm in methanol) cortisol, two cells with 10 ng cortisol and finally two cells with 20 ng cortisol. All the cells were spiked with an IS level of 20 ng (200 μL 0.1 ppm d_4_-cortisol in methanol).

The analytical procedure was performed as described in the main paper (under Matrials and methods). and the cortisol concentration (ng/g) was determined to be 16.3 ng/g (dw) (9 replicates) and 13.0 ng/g using an external standard curve (3 replicates) by use of standard addition. The signal to noise ratio of the cortisol peaks in the three non-spiked replicate samples ranged between 40-48, indicating that cortisol can indeed be quantified in harbor porpoises samples <300 mg dw skin. Instrument variations for samples containing skin tissue were 2.3% and 2.9% for d_4_-cortisol and cortisol, respectively.


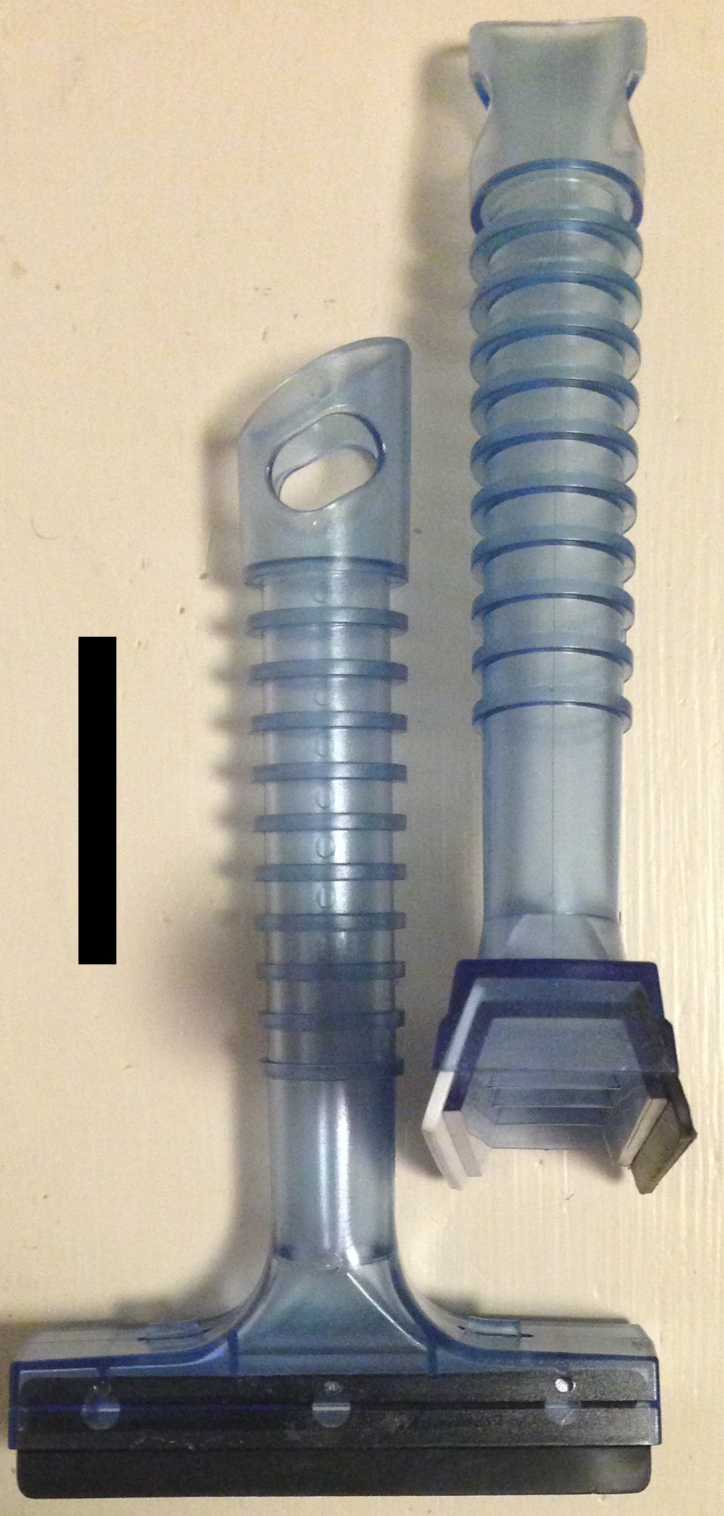


**Supplementary material Fig. 1**

The rubberized ice scraper (black, rubberized edge) deemed most efficient in comparison with other harbor porpoise (*Phocoena phocoena*) non-invasive skin sampling methods (please see text for further details). Product EAN number: 9002058645807. Bar: 5 cm. (Photo: TØB)

**
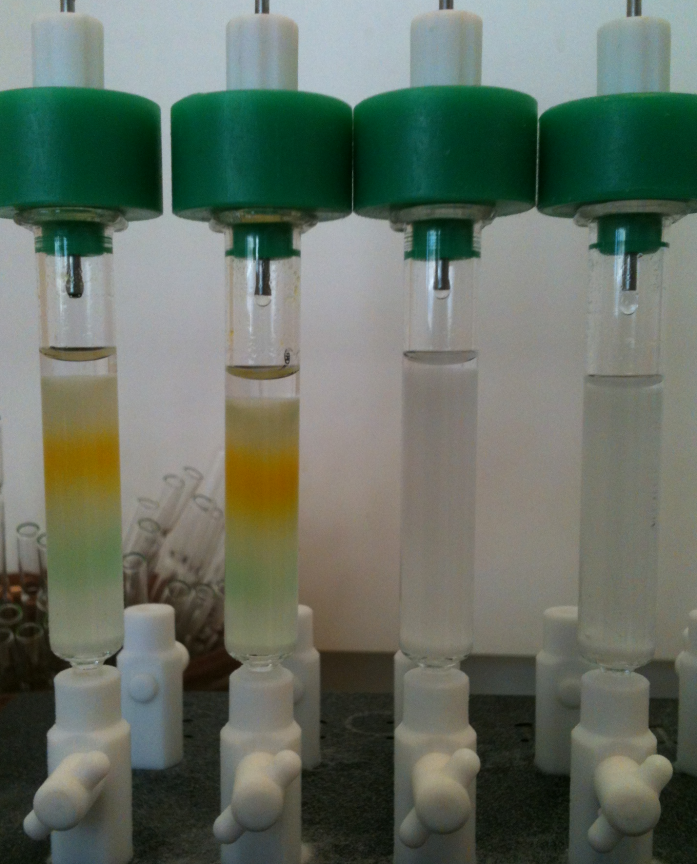
**

**Supplementary material Fig. 2**

Picture taken during the silica gel column clean-up part of the analytical method described in the manuscript text. Left: Right and left side scrape samples from harbor porpoise 47501 right. Right: Left and right side scrape samples from harbor porpoise 2012-60022. The visible difference in amount of co-extracted compound between the two animals suggests that the scrapes of porpoise 47501 contained material other than just epidermal cells. (Photo: JJW).

**Supplementary material Fig. 3**

Chromatograms of ion transmissions for d_4_-cortisol and cortisol peaks from harbor porpoise (*Phocoena phocoena;* id: 43721) dorsal fin plugs 2 + 3, spiked with 20 ng d_4_-cortisol prior to extraction. Retention times (RT) were 28.51 minutes and 28.52 minutes for d_4_-cortisol and cortisol, respectively. cps: Counts per second.
